# Cerebral vascular malformations: pathogenesis and therapy

**DOI:** 10.1002/mco2.70027

**Published:** 2024-12-08

**Authors:** Qiheng He, Ran Huo, Yingfan Sun, Zhiyao Zheng, Hongyuan Xu, Shaozhi Zhao, Yang Ni, Qifeng Yu, Yuming Jiao, Wenqian Zhang, Jizong Zhao, Yong Cao

**Affiliations:** ^1^ Department of Neurosurgery Beijing Tiantan Hospital Capital Medical University Beijing China; ^2^ Basic and Translational Medicine Center China National Clinical Research Center for Neurological Diseases Beijing China; ^3^ Research Unit of Accurate Diagnosis Treatment, and Translational Medicine of Brain Tumors Chinese Academy of Medical Sciences and Peking Union Medical College Beijing China Beijing China; ^4^ Department of Neurosurgery Peking Union Medical College Hospital Chinese Academy of Medical Sciences and Peking Union Medical College Beijing China Beijing China; ^5^ Collaborative Innovation Center Beijing Institute of Brain Disorders Beijing China

**Keywords:** arteriovenous malformations, biopsy, cavernous malformations, cerebral vascular malformation, microenvironment, somatic mutation

## Abstract

Cerebral vascular malformations (CVMs), particularly cerebral cavernous malformations and cerebral arteriovenous malformations, pose significant neurological challenges due to their complex etiologies and clinical implications. Traditionally viewed as congenital conditions with structural abnormalities, CVMs have been treated primarily through resection, embolization, and stereotactic radiosurgery. While these approaches offer some efficacy, they often pose risks to neurological integrity due to their invasive nature. Advances in next‐generation sequencing, particularly high‐depth whole‐exome sequencing and bioinformatics, have facilitated the identification of gene variants from neurosurgically resected CVMs samples. These advancements have deepened our understanding of CVM pathogenesis. Somatic mutations in key mechanistic pathways have been identified as causative factors, leading to a paradigm shift in CVM treatment. Additionally, recent progress in noninvasive and minimally invasive techniques, including gene imaging genomics, liquid biopsy, or endovascular biopsies (endovascular sampling of blood vessel lumens), has enabled the identification of gene variants associated with CVMs. These methods, in conjunction with clinical data, offer potential for early detection, dynamic monitoring, and targeted therapies that could be used as monotherapy or adjuncts to surgery. This review highlights advancements in CVM pathogenesis and precision therapies, outlining the future potential of precision medicine in CVM management.

## Graphical abstract

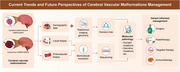

## 1 INTRODUCTION

Cerebral vascular malformations (CVMs) are a heterogeneous group of disorders characterized by abnormal vascular development in the brain. The most common types of CVMs include cerebral arteriovenous malformations (AVMs) and cerebral cavernous malformations (CCMs).^1^, ^2^ Cerebral AVMs are complex tangles of abnormal blood vessels where arteries connect directly to veins, bypassing the normal capillary network.^3^ This direct connection causes high‐pressure arterial blood flow into the low‐pressure venous system, increasing the risk of vessel rupture and hemorrhage.^1^, ^4^ AVMs can occur sporadically or in association with genetic syndromes such as hereditary hemorrhagic telangiectasia.^5^ Diagnosis typically involves imaging techniques like magnetic resonance imaging (MRI) and digital subtraction angiography. The prevalence of cerebral AVMs is approximately 1 in 100,000 individuals, with an annual detection rate of 1–1.8 per 100,000 person‐years.^6^, ^7^ AVMs are most commonly detected in young adults, often between the ages of 20 and 40 years.^8^ The clinical impact of AVMs is significant due to their potential to cause life‐threatening intracerebral hemorrhages, seizures, and progressive neurological deficits. The overall annualized bleeding rate is 3.0%, with 2.2% for unruptured AVMs and 4.5% for ruptured AVMs.^9^ Hemorrhages from AVMs have a mortality rate of approximately 10–15% and a morbidity rate of 20–30%, underscoring the need for timely diagnosis and intervention. Retrospective data indicate that hemorrhage occurs more commonly in children than in adults at initial presentation (56 vs. 43%).^10^, ^11^

CCMs are structurally abnormal, slow‐flowing collections of capillaries primarily found in the central nervous system. These malformations consist of multiple mulberry‐like cavities formed by dilated thin‐walled capillaries devoid of normal brain parenchyma. CCMs affect approximately 0.16–0.9% of the general population, with an estimated annual detection rate of 0.56 per 100,000 person‐years among adults. The prevalence of CCMs increases with age.^12^, ^13^, ^14^, ^15^, ^16^, ^17^, ^18^, ^19^, ^20^ Most CCM cases are sporadic and solitary, accounting for 80–90% of cases, while familial cases comprise 10–20%. Radiation‐induced CCMs are also becoming more common.^13^, ^21^, ^22^, ^23^, ^24^, ^25^ The widespread availability of cerebral imaging has led to higher detection of CCMs, making it more likely that physicians will encounter patients with these lesions.^15^ CCM symptoms vary widely, ranging from incidental findings to seizures, focal neurological deficits, and intracerebral hemorrhages.^22^, ^26^ Notably, 70% of patients with CCMs are asymptomatic.^12^, ^19^ Although the risk of hemorrhage is lower than that with AVMs, recurrent bleeding can still lead to significant morbidity.

Recent studies have identified somatic mutations in endothelial cells (ECs) from AVMs and CCMs using deep gene sequencing of surgical specimens. These mutations have been shown to induce CVMs‐like abnormalities in mouse models, suggesting a shift in the understanding of CVM pathogenesis from simple vascular structural dysplasia to gene mutations that drive vascular diseases.

Significant advancements in understanding disease mechanisms are expected to lead to revolutionary treatment approaches. This review discusses these developments, focusing on the evolving landscape of CVM diagnosis and treatment and emphasizing the need for further research and integrating genetic findings into clinical practice to improve patient outcomes.

## 2 CVMs FROM THE PERSPECTIVE OF CONGENITAL VASCULAR ANOMALIES CHARACTERIZED BY STRUCTURAL LESIONS

CVMs have traditionally been viewed as congenital vascular anomalies characterized by structural lesions. Early studies emphasized their congenital nature, attributing their development to embryonic vascular dysregulation. Current treatment strategies primarily focus on physically eliminating these malformations through surgical resection, embolization, or stereotactic radiosurgery (SRS). Although these methods aim to eliminate abnormal vessels, they often involve significant risks of neurological damage due to their invasive nature.

### 2.1 Primary surgical techniques

The primary treatment modalities for CVMs include surgical resection, embolization, and SRS. Surgical resection entails the direct removal of the malformation via craniotomy, often preferred for accessible AVMs and CCMs that cause significant symptoms or pose a high risk of hemorrhage.^1^, ^27^, ^28^, ^29^ However, surgical intervention carries a risk of neurological damage, particularly for malformations located in eloquent brain areas.^30^, ^31^ Recent advancements in intraoperative imaging and neuronavigation have enhanced the precision of surgical resection and reduced the risk of neurological deficits.

Embolization is a minimally invasive procedure that injects embolic agents into abnormal blood vessels to occlude them. This technique is frequently used as a preoperative adjunct to decrease the size of AVMs or as a standalone treatment for smaller malformations. While embolization can effectively reduce AVM size, it may not eliminate the malformations, necessitating additional therapies and increasing the risk of vessel rupture, as indicated by a subgroup analysis from the TOBAS trial.^32^, ^33^ Furthermore, incomplete embolization may elevate the risk of AVM bleeding.^33^, ^34^ CCMs, which are angiographically occult, are not amenable to endovascular approaches.

SRS has emerged as a noninvasive alternative for AVM treatment, delivering focused radiation beams to induce gradual occlusion of abnormal vessels. This method is beneficial for deep‐seated or surgically inaccessible malformations. Although SRS has a lower immediate risk than surgery, the effects of radiation may take 2–3 years to manifest, with complications accumulating over time. While the SRS obliteration rate of SRS is approximately 70%,^35^, ^36^ there is a potential risk of radiation‐induced complications, including delayed cyst formation and vasculopathy issues.^37^, ^38^, ^39^, ^40^ The use of SRS for CCMs is controversial due to the presence of hemosiderin, a radiation sensitizer that can significantly increase the risk of complications.^41^, ^42^, ^43^, ^44^ Despite advancements in these techniques, the primary goal remains the physical destruction of the CVMs.

### 2.2 Evolution from life‐saving to function‐preserving treatments

While these treatments aim to save lives by preventing hemorrhage, improving quality of life has become an equally important objective. This shift has led to the development and implementation of new technologies aimed at preserving neurological function before, during, and after surgical interventions. Advances in techniques such as functional MRI (fMRI), diffusion tensor imaging (DTI), intraoperative neuronavigation, and intraoperative Doppler ultrasound enable surgeons to accurately delineate malformation boundaries and avoid critical functional areas, thus reducing the risk of postoperative neurological deficits.

fMRI allows for mapping critical brain functions, such as motor and language areas, before surgery with high temporal and spatial resolutions. This preoperative evaluation assists in planning the surgical approach to avoid critical regions and minimize functional deficits.^45^ Wang et al.^46^ compared the results of electrical cortical stimulation and fMRI in 43 patients with AVMs, demonstrating that fMRI exhibits high sensitivity for motor mapping.

DTI and fiber bundle tracking create spatial images by calculating the anisotropy of water molecules using MRI, which facilitates fiber tracking.^47^ This technique aids in defining the lesion‐to‐eloquence distance (LED), the distance between the lesion and the nearest eloquent area. Jiao et al.^48^ found that LED is an essential predictor of preoperative risk assessment in patients with AVM, developing the HDVL grading system that provides a detailed evaluation of vascular malformations. This system includes hemorrhagic presentation, diffuseness, deep venous drainage, and LED. It is more accurate than the Spetzler‐Martin grading system in predicting surgical outcomes. This comprehensive evaluation assists in surgical planning and risk assessment, ensuring a more targeted and effective intervention.^49^, ^50^

Intraoperative Doppler ultrasonography can detect residual or small CCM lesions. This technique provides immediate feedback on the hemodynamic status of the vessels involved in the malformation, enabling the surgeon to make informed decisions regarding resection. Enhanced contrast in ultrasound angiography can help identify superficial and deeper arterial blood vessels.^51^, ^52^ However, ultrasound may miss areas of slow blood flow or very small blood vessels (< 0.6 mm in diameter).^53^

Intraoperative neuronavigation systems offer real‐time guidance during surgery, allowing for precise localization and resection of malformations. By integrating preoperative CT or MRI data, neuronavigation enhances surgical accuracy and reduces the risk of neurological damage. Winter et al.^54^ found that combining neuronavigation with fMRI significantly improved resection volume, surgical duration, and neurological outcomes in patients with supratentorial CCMs. Mathiesen et al.^55^ proposed an intraoperative imaging and navigation system that utilizes intraoperatively acquired three‐dimensional ultrasound data alongside preoperative MRI scans and MR angiograms, which demonstrated improved outcomes in managing AVM pathological vessels.

Postoperative rehabilitation programs tailored to individual patient needs play a vital role in restoring and optimizing neurological function. These programs may include physical therapy, acupuncture, and electrical nerve stimulation.^56^, ^57^, ^58^, ^59^

Despite these advancements, the fundamental principle of CVM treatment remains the physical destruction or removal of abnormal vascular structures. This underscores the necessity for alternative therapeutic approaches to address the underlying causes of these malformations.

## 3 EXPLORATION OF NEW PATHOGENESIS OF CVMs

Advances in next‐generation sequencing (NGS) technology have uncovered mutations associated with CVMs, and recent discoveries in the molecular and genetic underpinnings of CVM have revolutionized our understanding of their pathogenesis.^60^ The identification of endothelial somatic and de novo germline mutations in AVMs and CCMs has provided critical insights into the mechanisms driving these malformations.^61^ These findings suggest that CVMs are not merely congenital anomalies but the result of complex genetic and molecular interactions.

### 3.1 Genetic mutations in CVMs

#### 3.1.1 Genetic mutations in AVMs

Germline and somatic mutations play pivotal roles in the development of AVMs. Specifically, somatic mutations in genes such as KRAS and BRAF have been implicated as key contributors to these vascular anomalies^62^ (Table 1). In 2018, Nikolaev et al.^63^ identified EC‐specific KRAS mutations (KRAS^G12V^, KRAS^G12D^, and KRAS^Q61H^) in lesion tissues from 60% of patients with AVM, with no corresponding mutations detected in their blood samples. Hong et al.^64^ confirmed a high frequency of KRAS somatic mutations within AVM lesions, evidenced in 16 of 21 cases examined. However, subsequent studies reported varying frequencies of somatic KRAS mutations in AVMs, ranging from 28.5 to 76.2%.^63^, ^64^, ^65^, ^66^, ^67^, ^68^ Detection of this mutation was limited in these studies due to sequencing depth and the number of cases included. In 2021, Li et al.^69^ established a large‐scale AVM cohort and found that out of 179 cases, 129 exhibited somatic KRAS mutations associated with hemorrhage as the initial symptom. Hong proposed that BRAF mutations downstream of KRAS also occur in AVM ECs.^64^ Similarly, Goss et al.^67^ validated corresponding somatic BRAF mutations. This suggests that mutations in KRAS and BRAF in ECs are critical molecular events and hallmark features of AVM pathogenesis.

**TABLE 1:** Mutations related to sporadic AVM.

Genes	Affected/total (%)	Sample	Technique	Variant	Affected/total (%)	References
KRAS	12/26	Flash‐frozen AVM tissue, blood sample	WES	c.35G>A p.Gly12Asp	8/26	Nikolaev et al.^63^
				c.35G>T p.Gly12Val	4/26	
	29/39	Flash‐frozen AVM tissue, blood sample	ddPCR	c.35G>A p.Gly12Asp	19/39	
				c.35G>T p.Gly12Val	9/39	
				c.183A>T p.Gln61His	1/39	
	16/33	Paraffine‐embedded AVM tissue, blood sample	ddPCR	c.35G>A p.Gly12Asp	12/33	
				c.35G>T p.Gly12Val	4/33	
KRAS	16/21	Fresh AVM tissue, whole blood sample	TS, ddPCR	c.35G>A p.Gly12Asp	11/21	Hong et al.^64^
				c.35G>T p.Gly12Val	4/21	
				c.191_196dupACAGTG	1/21	
				p.S65_Ala66insAspSe		
KRAS	25/38	Paraffine‐embedded of AVM tissue	ddPCR, Immunohistochemistry	c.35G>A p.Gly12Asp	15/38	Oka et al.^66^
				c.35G>T p.Gly12Val	10/38	
KRAS	8/16	Flash‐frozen AVM tissue, blood sample	MIP‐seq, ddCPR	c.35G>A p.Gly12Asp	5/16	Goss et al.^67^
				c.35G>T p.Gly12Val	3/16	
KRAS	6/21	Paraffine‐embedded AVM tissue	PCR	c.35G>T p.Gly12Val	5/21	Priemer et al.^65^
				c.34G>T p.Gly12Cys	1/21	
KRAS	129/179	Flash‐frozen AVM tissue, whole blood sample	WES, amplicon sequencing, ddPCR	c.35G>A p.Gly12Asp; c.35G>T p.Gly12Val; c.35G>C p.Gly12Ala; G12S c.34G>A, p.Gly12Ser;c.183A>T p.Gln61His	129/179	Li et al.^69^
KRAS	6/14	Flash‐frozen AVM tissue, blood sample	WES, ddPCR	c.35G>A p.Gly12Asp	4/14	Gao et al.^68^
				c.35G>T p.Gly12Val	2/14	
	31/56	Flash‐frozen AVM tissue, blood sample	ddPCR	c.35G>A p.Gly12Asp/c.35G>T p.Gly12Val	31/56	
BRAF	1/21	Fresh AVM tissue, whole blood sample	TS, ddPCR	c.1799T>A p.Val600Glu	1/21	Hong et al.^64^
BRAF	2/16	Flash‐frozen AVM tissue, blood sample	MIP‐seq, ddCPR	c.1799T>A p.Val600Glu	1/16	Goss et al.^67^
				Q636X	1/16	
SIRT	1/1	Flash‐frozen AVM tissue, blood sample	WES, Sanger sequencing.	g.67884831C>T c.110C>T p.Pro37Leu	1/1	Mukhtarova et al.^70^
SMAD9	1/1	AVM tissue, blood sample	WES, Sanger sequencing	c.C739T p.R247X	1/1	Walcott et al.^71^

Abbreviations: ddPCR, droplet digital polymerase chain reaction; MIP‐seq, molecular inversion probe sequencing; TS, targeted sequencing; WES, whole‐exome sequencing.

Gao et al.^68^ identified additional candidate somatic mutations linked to sporadic AVMs, such as PDGFRB and CRKL, through whole‐exome sequencing (WES) of AVM tissues and blood DNA. Case reports have also highlighted rare mutations, including SIRT1 and SMAD9, prompting further exploration and functional analysis to enhance our understanding of the biological underpinnings of AVM.^70^, ^71^

WES studies of AVMs have identified several rare germline mutations. WES of blood samples from five patients with AVM indicated that germline mutations in genes such as FBN2, TAB1, NCoR2, SLIT2, RNF111, CAMK2B, EPHA2, and EPHB2 may be associated with vascular homeostasis.^72^ Additionally, Li discovered de novo germline mutations in AVMs, including splicing mutations in ENG, stop codon mutations in JUP, and mutations in EXPH5 and CHEK2, which often exhibit mutual exclusivity with KRAS mutations.^69^

#### 3.1.2 Genetic mutations in CCMs

CCMs can be classified as sporadic, familial, or radiation‐induced cavernous malformations (RICMs).^73^, ^74^, ^75^ Familial CCMs are inherited in an autosomal dominant manner and present as multiple lesions. Patients with familial CCMs exhibit biallelic germline and somatic loss‐of‐function (LOF) mutations in one of three CCM genes: Krev interaction trapped 1 (KRIT1/CCM1), CCM2, and programmed cell death 10 (PDCD10/CCM3). These mutations disrupt endothelial homeostasis and proliferation, contributing to the pathogenesis of familial CCMs.^73^, ^76^, ^77^, ^78^ Sporadic CCMs may present as a single de novo lesion, with or without a venous anomaly, and are usually asymptomatic and nonhereditary.^79^, ^80^, ^81^ Additionally, somatic mutations in the CCM1/CCM2/CCM3 signaling complex have been confirmed in sporadic CCM^82^, ^83^ (Table 2). However, Cao et al.^84^, ^85^ discovered that in sporadic CCM, somatic mutations in the CCM1/2/3 genes account for only 19.4% of cases, with MAP3K3 mutations present in 37% of cases; notably, these two are mutually exclusive. The role of MAP3K3 mutations in both cerebral and spinal cord cavernous malformations is noteworthy.^86^ Unlike AVMs, CCMs with a single CCM gene or a MAP3K3 mutation tend to remain stable and nonprogressive, suggesting that other mutations or factors may trigger vascular proliferation and pathological progression in CCMs, resulting in a “two hit” effect.^87^

**TABLE 2:** Mutations related to sporadic CCM.

Genes	Affected/total (%)	Sample	Technique	Variant	Affected/total (%)	References
KRIT1 (CCM1)	2/11	CCM tissue	TS, Sanger sequencing	c.1659_1688delins	1/11	McDonald et al.^82^
				c.993T.G, p.Y331X; c.1159C.T, p.Q387X	1/11	
KRIT1 (CCM1)	15/79	Flash‐frozen CCM tissue	ddPCR, TS, single‐nucleus DNA sequencing		15/79	Ren et al.^83^
CCM2	2/11	CCM tissue	TS, Sanger sequencing	c.355_369del	1/11	McDonald et al.^82^
				c.611_622del	1/11	
CCM2	5/79	Flash‐frozen CCM tissue	ddPCR, TS, single‐nucleus DNA sequencing		5/79	Ren et al.^83^
PDCD10 (CCM3)	1/79	Flash‐frozen CCM tissue	ddPCR, TS, single‐nucleus DNA sequencing		1/79	Ren et al.^83^
CCM1/CCM2/CCM3	21/94	CCM tissue, whole blood sample	WES, ddPCR		21/94	Huo et al.^84^
MAP3K3	34/92	Fresh‐frozen CCM Tissue	WES, ddPCR	c.1323C>G p.Ile441Met	34/92	Weng et al.^85^
MAP3K3	42/73	Fresh‐frozen CCM Tissue	WES, ddPCR	c.1323C>G p.Ile441Met	42/73	Hong et al.^89^
MAP3K3	82/94	CCM tissue, whole blood sample	WES, ddPCR	c.1323C>G p.Ile441Met	82/94	Huo et al.^84^
PIK3CA	56/79	Flash‐frozen CCM tissue	ddPCR, single‐nucleus DNA sequencing	c.1258T>C p.Cys420Arg	3/79	Ren et al.^83^
				c.1357G>A p.Glu453Lys	1/79	
				c.1624G>A p.Glu542Lys	19/79	
				c.1633G>A p.Glu545Lys	13/79	
				c.3145G>C p.Gly1049Arg	2/79	
				c.3140A>G p.His1047Arg	15/79	
				c.3127A>G p.His1047Arg	2/79	
				c.1035T>G p.Asn345Lys	1/79	
PIK3CA	45/73	Fresh‐frozen CCM Tissue	WES, ddPCR	c.1258T>C p.Cys420Arg	6/73	Hong et al.^89^
				c.1624G>A p.Glu542Lys	5/73	
				c.1633G>A p.Glu545Lys	14/73	
				c.3140A>G p.His1047Arg	21/73	
PIK3CA	51/94	CCM tissue, whole blood sample	WES, ddPCR	c.3140A>G p.His1047Arg	19/94	Huo et al.^84^
				c.1624G>A p.Glu542Lys	19/94	
				c.1633G>A p.Glu545Lys	6/94	
GJA4	21/58	Fresh CCM Tissue, blood sample, paraffin‐embedded CCM tissue	WES, ddPCR	c.121G>T p.Gly41Cys	21/58	Huo et al.^84^
GJA4	1/31	Paraffin‐embedded CCM tissue and fresh‐frozen CCM tissue	WES, TS, PCR	c.121G>T p.Gly41Cys	1/31	Ren et al.^94^
GNA14	14/31	Paraffin‐embedded CM tissue and fresh‐frozen CCM tissue	WES, TS, PCR	c.614A>T p.Gln205Leu	14/31	Ren et al.^94^

Abbreviations: ddPCR, droplet digital polymerase chain reaction; PCR, polymerase chain reaction; TS, targeted sequencing; WES, whole‐exome sequencing.

In 2021, Ren et al.^83^ reported that 71% of CCMs contain hotspot‐activating mutations in PIK3CA. This finding was corroborated in subsequent studies, indicating a somatic mutation rate of PIK3CA ranging from 28.9 to 71%.^83^, ^85^, ^88^, ^89^ Cao et al.^84^ established the largest Chinese cohort of sporadic CCM, where among 94 patients with CCM, 44 had CCM1/CCM2 or MAP3K3 mutations, with 75% also having concurrent PIK3CA mutations. This revealed a high degree of coexistence among CCM mutations, MAP3K3, and PIK3CA mutations. In contrast to CCM lesions in neonatal mice induced by MAPK3 mutations, which gradually disappeared with age, the CCM model induced by the combined mutations of MAP3K3 and PIK3CA promoted the formation of CCM and increased lesion count. Further follow‐up showed a stable number of lesions generated by the cooperative effect of the PIK3CA gain‐of‐function (GOF) after 3 months, highlighting that PIK3CA mutations are necessary synergistic factors in patients with CCM. This is consistent with the hypothesis proposed by Kahn, which posits that when a lesion in a patient with CCM or MAP3K3 gene mutations acquires a PIK3CA somatic mutation during disease progression, the lesion enlarges, leading to recurrent hemorrhaging.^90^ This phenomenon was further confirmed by Hong et al.^89^ in a prospective cohort study of 73 patients with sporadic CCM, where lesions with PIK3CA mutations exhibited a significant increase in hemorrhage volume on imaging, suggesting a critical role for this mutation in CCM‐related hemorrhage.

Patients with RICMs often have a history of undergoing cranial radiotherapy. The etiology is believed to involve direct radiation‐induced damage and necrosis of cerebral blood vessels, accompanied by a delayed response of new blood vessels, leading to CCMs. Some researchers attribute this type of disease to sporadic mutations.^74^, ^91^, ^92^

Huo et al.^93^ were the first to discover that over one‐third of extra‐axial cavernous hemangioma (ECH) tissue samples contained EC‐specific somatic GJA4 mutations. They observed that lesions with these mutations predominantly exhibited mulberry‐like lesions on contrast‐enhanced MRI.^93^ Furthermore, in an analysis of somatic mutations in 31 patients with ECH, Ren et al.^94^ reaffirmed the role of GJA4 mutations and identified GNA14 mutations in one‐third of the patients with cavernous sinus hemangiomas. Notably, in these samples, the GNA14 and GJA4 mutations were mutually exclusive, a mechanism that requires further elucidation (Table 3).

**TABLE 3:** Mutations related to ECH.

Genes	Affected/total (%)	Sample	Technique	Variant	Affected/total (%)	References
GJA4	5/12	Fresh‐frozen or Paraffine‐embedded ECH tissue, blood	WES, ddPCR	c.121G>T, p.G41C	5/12	Huo et al.^84^
	16/46	Paraffine‐embedded ECH tissue	ddPCR	c.121G>T, p.G41C	16/46	
GJA4	1/3	Fresh‐frozen or Paraffine‐embedded cavernous sinus hemangiomas tissue, blood	WES, TS	c.121G>T, p.G41C	1/3	Ren et al.^94^
GNA14	1/3	Fresh‐frozen or Paraffine‐embedded cavernous sinus hemangiomas tissue, blood	WES, TS	c. 614A>T p.Gln205Leu	1/3	

Abbreviations: ddPCR, droplet digital polymerase chain reaction; MIP‐seq, molecular inversion probe sequencing; TS, targeted sequencing; WES, whole‐exome sequencing.

### 3.2 Mechanisms of mutation‐induced pathogenesis

#### 3.2.1 Mechanism in AVM

Recent observations of EC‐specific KRAS and BRAF mutations in AVMs suggest that the RAS/RAF/MAPK pathway significantly contributes to disease pathogenesis.^95^ Nikolaev et al.^63^ proposed that KRAS mutations could activate the MAPK/ERK pathway, leading to angiogenesis, cell migration, and proliferation.^96^ Mouse and zebrafish models further confirm that KRAS mutations enhance AVM progression through the MEK/ERK pathway rather than PI3K signaling.^97^, ^98^ KRAS mutations can also induce endothelial‐to‐mesenchymal transition (EndMT), a crucial process in AVM development.^69^ Xu et al.^99^ suggested that the TGF‐β/BMP–SMAD4 pathway mediates this process, while the knockdown of SMAD4 can reverse EndMT. Similar to KRAS, BRAF is a well‐established oncogene. Studies have shown that the BRAF^V600E^ mutation induces typical pathological features of AVM in mice, including vascular wall instability, dilation, focal intracranial hemorrhage, and an inflammatory microenvironment.^100^ These effects are thought to be mediated through the MAP pathway and its downstream signal transduction. However, the specific underlying mechanisms warrant further investigation. The neurovascular unit (NVU) is a complex composed of neurons, glial cells (including microglia, astrocytes, and oligodendrocytes), and vascular components (pericytes, ECs, and smooth muscle cells). Studies indicate that pericytes in the NVU are significantly reduced in AVM, and the Notch or PDGFB pathways contribute to severe AVM by disrupting pericyte homeostasis.^101^, ^102^, ^103^, ^104^ ECs also play a crucial role in the NVU. Studies have shown that MHC class II molecules are abnormally expressed in AVMs, potentially affecting interactions with immune cells.^101^

**FIGURE 1:**
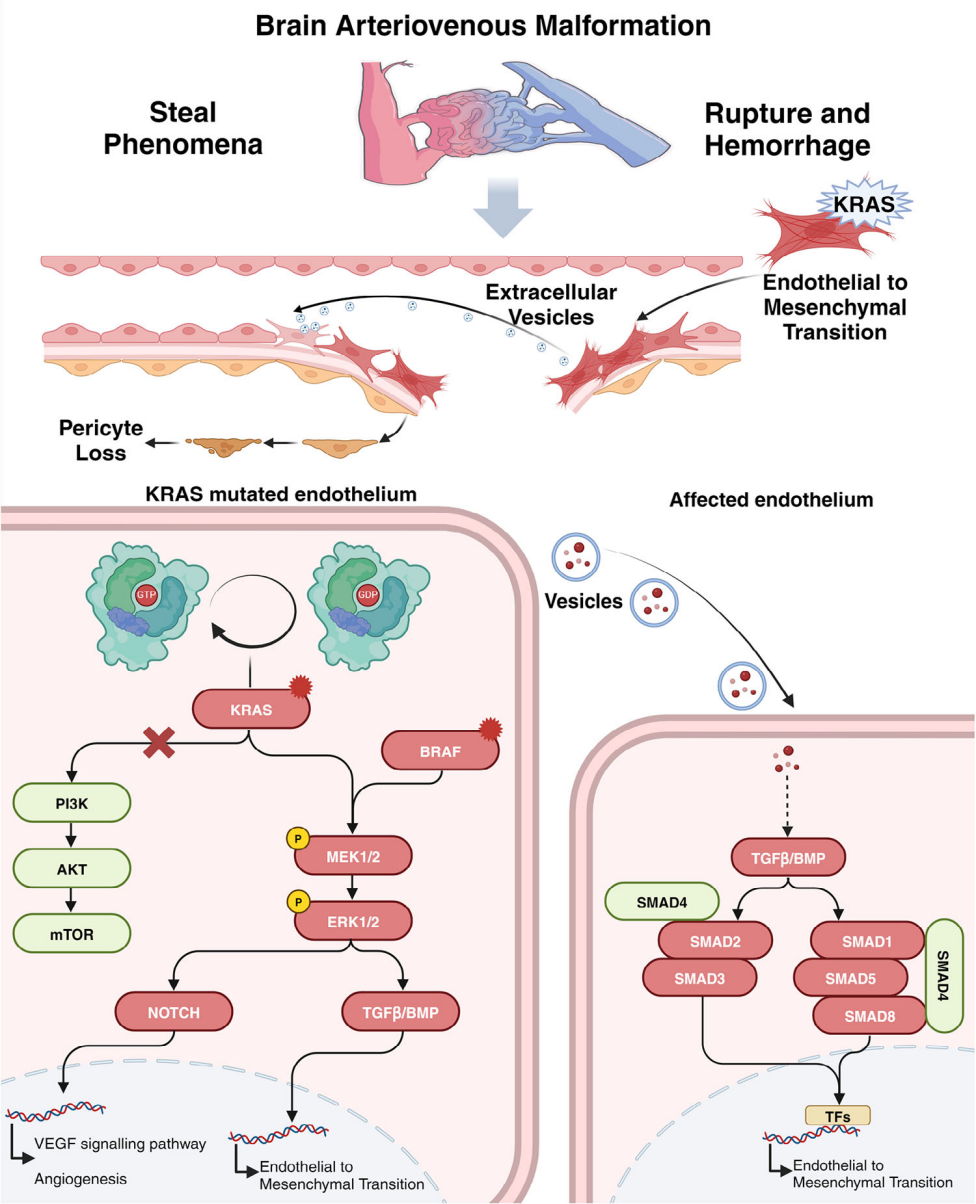
Mechanisms of mutation‐induced pathogenesis in sporadic AVMs. KRAS mutated endothelium promotes the occurrence and development of AVMs through a variety of ways, including promoting EndMT, angiogenesis, VEGF signaling pathway and pericyte loss. Kras mutation in endothelial cells activates the MEK/ERK pathway, but has no direct effect on PI3k. Subsequently, KRAS/BRAF activates the Notch pathway or TGFb/BMP pathway through the MEK/ERK pathway, thereby affecting endothelial cell function. It is worth noting that these mutated endothelial cells also release vesicles that affect normal endothelial cells. Created with BioRender.com.

Although KRAS mutations in ECs of AVM are infrequent, widespread pathological changes in the endothelium indicate that low‐frequency KRAS mutations may amplify signaling by disturbing cellular communication and facilitating interactions between ECs and other cell types. He et al.^105^ found that ECs with the KRAS^G12D^ mutation release exosomes, which promote EndMT in adjacent normal ECs and induce extensive vascular remodeling. This discovery reveals the communication between mutated and normal endothelium in AVMs and offers new strategies for further investigating AVM progression (Figure 1).

#### 3.2.2 Mechanism in CCM

By constructing animal models with corresponding mutations, it has been demonstrated that mutations in the CCM1/2/3, MAP3K3, and PIK3CA genes are pathogenic for CCM.^83^, ^84^, ^86^, ^106^, ^107^, ^108^ Several mechanistic studies have established the MEKK3–KLF2/4 signaling pathway as a central signaling axis in CCM disease pathogenesis. Additionally, various auxiliary factors contribute to CCM development.

Genomic studies of patients with CCM have identified LOF mutations in CCM1/2/3 within CCM lesions. Systemic gene mutations in animal models indicate that CCM genes are essential for normal vascular development in zebrafish and mice.^109^, ^110^, ^111^, ^112^ Subsequent studies using endothelial‐specific CCM mutant mouse models confirmed that CCM LOF mutations are causative factors for CCM.^106^, ^107^, ^108^ Protein interaction studies revealed that the CCM complex interacts with MEKK3 through CCM2, inhibiting MEKK3 under normal conditions.^113^, ^114^ When one of the CCM trimer subunits undergoes an inactivating mutation, its inhibitory effect on MEKK3 is reversed, activating the MEKK3 signaling pathway. In endothelial‐specific CCM LOF mouse models, the knockout of MEKK3 or its downstream transcription factors, KLF2 or KLF4, is sufficient to prevent CCM formation.^115^, ^116^, ^117^ Mice with KLF4 GOF mutations develop typical CCM lesions.^83^ These studies established the MEKK3–KLF2/4 signaling pathway as the central axis in CCM pathogenesis.

The PIK3CA pathway is another crucial pathway involved in CCM formation and development. Recent genetic studies on patients with CCM found that most CCM lesions also carry PIK3CA mutations,^83^, ^84^ and single‐nucleus sequencing indicates that PIK3CA mutations coexist with CCM mutations in the same cells.^118^ This suggests that the PI3K pathway plays a significant role in CCM pathogenesis. Additionally, studies on CCM mouse models have shown that lesions are more likely to form postnatally in areas with active angiogenesis, such as the cerebellum and retina.^119^ The earlier the CCM gene is knocked out, the more pronounced the CCM phenotype.^120^ Adding the angiogenic growth factor VEGF‐A or losing the antiangiogenic factor thrombospondin 1 (THBS1, TSP1) increases CCM formation.^121^, ^122^ These findings suggest that CCM development requires an environment conducive for EC proliferation, with PIK3CA mutations likely providing proliferative signals to ECs. Further animal model studies have shown that PIK3CA mutations exacerbate lesions in CCM and MAP3K3 mutant mouse models, and the use of the mTOR inhibitor rapamycin can inhibit CCM formation.^83^ Due to the unique mechanism of MAP3K3–I441M, lesions with only MAP3K3 mutations cannot persist stably; however, when combined with PIK3CA mutations, lesions can remain stable for extended periods.^84^ These studies demonstrate the crucial role of PIK3CA mutations and the PI3K–mTOR pathway in CCM.

**FIGURE 2:**
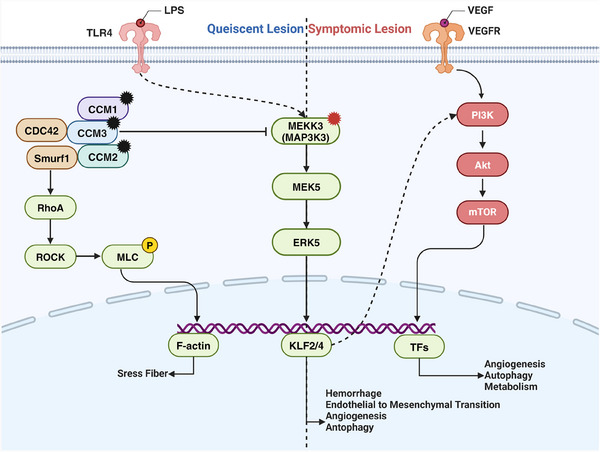
Mechanisms of mutation‐induced pathogenesis in sporadic CCMs. The inactivation mutation of CCM1/2/3 complex leads to a reduced inhibitory effect on MAP3K3. However, only with excessive activation of proliferation signals such as PI3K can clinical observations of CCM lesion enlargement or bleeding occur. Created with BioRender.com.

In addition to the critical MEKK3 and PI3K pathways, various synergistic factors contribute to CCM development. The pathological processes of CCM formation, growth, and hemorrhage are primarily related to signaling abnormalities in the NVU, including abnormal proliferative angiogenesis, blood–brain barrier hyperpermeability, inflammatory and immune‐mediated processes, anticoagulant vascular domains, and gut microbiota‐driven mechanisms.^123^, ^124^, ^125^ Studies have identified a “gut–brain axis” in CCM mouse models, where gram‐negative bacteria in the gut can secrete lipopolysaccharides that activate the MEKK3 pathway through EC TLR4 receptors, exacerbating CCM lesions.^126^, ^127^ Correlation studies between the gut microbiome and CCM lesion size and single nucleotide polymorphisms in TLR4 and CD14 in patients with CCM have validated this conclusion. In the CCM3 mouse model, B cell knockout significantly reduced lesion size and perilesional hemorrhage, indicating that B cells and humoral immunity also promote CCM^128^ (Figure 2).

In ECH, GJA4 mutations activate the SGK‐1 signaling pathway, upregulating critical genes involved in excessive cell proliferation and loss of arterial characteristics.^93^, ^94^ Furthermore, GNA14 mutations lead to the upregulation of the MAPK pathway and pathways related to angiogenesis.

## 4 ADVANCES IN IMAGING GENOMICS AND LIQUID BIOPSY IN CVMs

The advent of imaging genomics and liquid biopsy technologies has significantly enhanced the diagnostic and monitoring capabilities of CVMs. These advancements have shifted the focus from purely structural assessments to a more comprehensive understanding of the molecular and genetic landscapes of these malformations.

### 4.1 Imaging genomics

Imaging genomics integrates high‐throughput radiomics features and mathematical models to quantify CVM characteristics, linking imaging phenotypes with genetic, mutational, and expression patterns.^129^, ^130^ For example, integrating genomic data with MRI can reveal specific genetic mutations associated with distinct imaging phenotypes. AVMs with KRAS mutations, for instance, exhibit distinct imaging features that correlate with a higher propensity for hemorrhage. Similarly, CCMs with I441 M mutations display unique Zabramski grading patterns, informing prognosis and treatment planning.^85^

A landmark study used WES to identify somatic activating mutations in KRAS in brain AVMs.^63^ These mutations are found in many sporadic AVMs, providing a genetic basis for their formation. The study also showed that AVMs with KRAS mutations had distinct MRI characteristics, including increased flow voids and nidus size, and correlated with a higher risk of hemorrhage. This finding aligns with research on venous malformations, which suggests that PIK3CA‐ or TEK‐mutants activate the PI3K–Akt signaling pathway, leading to “slow‐flow” vascular malformations.^131^, ^132^ In contrast, “high flow” vascular anomalies, including sporadic brain and extracranial AVMs, are often linked with activating mutations in the RAS–MAPK pathway.^133^, ^134^, ^135^, ^136^ These imaging biomarkers may help identify AVMs with specific genetic profiles, improving diagnosis and personalized treatment planning. For extracranial vascular malformations, Stor et al.^137^ systematically reviewed 69 studies, finding that mutations in TIE2, PIK3CA, and PIK3R1 primarily manifested as small blue, low‐flow vascular malformations. Germline mutations in genes such as EPHB4 and RASA1 typically result in large pink/red lesions with high blood flow, suggesting a correlation between the imaging manifestations of vascular malformations and the associated gene mutations.^137^

Similarly, genomic imaging shows promise in the diagnosis and management of CCMs. Denier et al.^24^ and Gault et al.^138^ studied the relationship between CCM1/2/3 mutations and clinical phenotypes. Denier et al.^24^ reported that cerebral hemorrhage was the most common first symptom in patients with CCM3 mutations, and the increase in gradient‐echo sequence lesions with age differed between patients with CCM1 and CCM2 mutations.^24^ Gault et al.^138^ reported that patients with CCM1 mutations had fewer cases of bleeding unrelated to lesion size or number. Shenkar et al.^139^ demonstrated that CCM3 mutations lead to robust Rho‐associated protein kinase (ROCK) activity in murine and human CCM vasculature and increase brain vascular permeability in humans with CCM3 mutation. The clinical phenotype of CCM3 is more aggressive compared with familial and sporadic CCM1 and CCM2, with higher lesion burden and more frequent hemorrhages at an earlier age.^140^ Recent case series have also identified several clinical features in patients with CCM3 mutation.^141^, ^142^ Nikoubashman et al.^143^ utilized high‐resolution MRI combined with genomic sequencing to investigate the molecular underpinnings of familial CCMs. They found that specific CCM1 or CCM3 mutations were associated with distinct imaging patterns, such as multiple small lesions and a higher propensity for hemorrhage.^143^, ^144^ This integration of genomic and imaging data enhanced accurate disease severity assessment and progression, improving clinical management strategies for patients with familial CCMs. Interestingly, in children with CCM, the CCM2 genotype was linked to a higher risk of bleeding, and the greater number of lesions on MRI was a significant independent predictor of future symptomatic bleeding.^145^ Zabramski classified CCM lesions into four subtypes based on MRI findings. It was previously reported that Zabramski type IV is associated with germline mutations in CCM genes.^77^, ^146^ Wang et al.^147^ reported that the annual cumulative incidence of symptomatic hemorrhage in type I CCM was significantly higher than in types II and III in patients with sporadic CCM. Weng et al.^85^ detected MAP3K3 c.1323C>G mutation, predominately in type II or type III patients with CCM, defining a subclass of CCM. Choquet et al.^148^ investigated common variants in inflammatory and immune response genes that influence the severity of familial CCM1 disease. They found that IL‐4, CD14, IL‐6R, and MSR1 were associated with intracerebral hemorrhage and lesion counts on MRI, while TLR4, CD14, IL‐6R, and IGH were linked solely with lesion counts.

In animal models, Yang et al.^149^ employed the Dre–Cre dual recombinase system to specifically delete CCM genes in brain ECs, allowing for quantification of lesion burden in mice over time and enabling longitudinal drug testing in live animals. Similarly, Fisher et al.^150^ developed a chronic mouse model of CCM induced by postnatal ablation of CCM1 with Pdgfb–CreERT, discovering that MRI properties of lesions correlated with cellular markers for ECs, astrocytes, and microglia. These in vivo studies provide a foundation for understanding individual lesion characteristics and offer a comprehensive preclinical platform for testing new drugs and gene therapies for CCM control.

Artificial intelligence (AI) and machine learning (ML) algorithms are increasingly applied in imaging genomics to improve diagnostic accuracy and predict disease progression. These technologies can analyze vast datasets to identify subtle patterns and correlations that may not be apparent with conventional analyses, thus facilitating early detection and personalized treatment strategies. Saggi et al.^151^ used ML, including random forest models, gradient‐boosted decision trees, and AdaBoost, to predict the risk of hemorrhage in pediatric patients with AVMs who presented with or without hemorrhage and found left‐sided AVM, small AVM size, and concurrent arterial aneurysm as significant risk factors for hemorrhagic presentation. In adult patients with AVM, Oermann et al.^152^ pioneered the use of ML to predict outcomes after SRS, showing that ML models outperformed traditional scoring systems such as the Spetzler‐Martin grading scale, radiosurgery‐based AVM score, and Virginia Radiosurgery AVM Scale. Jiao et al.^153^ applied AI to predict postsurgical motor defects in patients with AVMs involving motor‐related areas. They found that FN_10mm/_50  mm can reflect the lesion‐fiber spatial relationship and be a key predictor of postsurgical motor defects in patients with brain AVMs. For CCM, existing ML models have primarily focused on predicting (re)bleeding events, leading to the development of various models.^154^, ^155^ Although radiological features have not yet been integrated with genomic data, AI‐driven genomic imaging has shown the potential to enhance diagnostic accuracy and guide personalized treatment strategies.^153^

### 4.2 Liquid biopsy

Liquid biopsy, a noninvasive technique involving blood or other body fluids analysis, offers a valuable tool for detecting and monitoring CVMs. Circulating biomarkers such as cell‐free DNA (cfDNA), noncoding RNAs (ncRNAs), proteins, and exosomes provide valuable insights into the genetic and molecular status of CVMs. cfDNA analysis can detect specific somatic mutations associated with CVMs, enabling early diagnosis and monitoring of disease progression^156^ (Table S1).

Zenner et al.^157^ used multiplex ddPCR to detect cfDNA in the plasma of patients with AVM, identifying cfDNA variants in two of eight cases. To circumvent the need for biopsy samples, Serio et al.^156^ performed molecular characterization of the peripheral blood of patients with AVM using cfDNA–NGS liquid biopsy and found KRAS mutations in 60% of patients with AVM. These findings suggest that cfDNA analysis could be a noninvasive diagnostic tool for early detection and disease monitoring in AVMs.

ncRNAs, including long ncRNAs (lncRNAs), microRNAs (miRNAs), small nucleolar RNAs (snoRNAs), and circular RNAs, are involved in cellular functions and homeostasis maintenance. However, they do not code for any functional proteins. These ncRNAs are thought to accurately reflect the human transcriptome and are stable and detectable in bodily fluids.^158^, ^159^ Li et al.^160^ examined lncRNA profiles in four pairs of AVM tissues and the corresponding superior temporal arteries or scalp artery fragments, discovering that certain lncRNAs are associated with seizures in patients with AVM. RNA sequencing in 10 patients with CCM and four controls revealed 1967 differentially expressed lncRNAs, with SMIM25 and LBX2‐AS1, found to correlate with more protein‐coding genes and implicated in critical signaling pathways, including vascular signaling and essential biological processes relevant to CCM pathophysiology.^161^ Research on miRNAs in CVMs has progressed further, with Huang et al.^162^ reporting decreased levels of miR‐137 and miR‐195* in AVM samples, both of which act as vasculogenic suppressors by altering phenotypic properties of smooth muscle cells in AVMs. Chen et al.^163^ identified miR‐7‐5p, miR‐199a‐5p, and miR‐200b‐3p as critical miRNAs involved in VEGF signaling in unruptured AVMs. Notably, miR‐135b‐5p was significantly upregulated in AVM and was associated with VEGF and HIF‐1α, which were further upregulated in ECs derived from patients with AVM when treated with hypoxia or VEGF.^164^

For CCMs, Kar et al.^165^ performed small RNA sequencing on CCM specimens and identified five key miRNAs relevant to CCM pathology (let‐7b‐5p, miR‐361‐5p, miR‐370‐3p, miR‐181a‐2‐3p, and miR‐95‐3p). Their miRNA–mRNA network analysis revealed functional relationships between the miRNAs and critical genes MIB1, HIF1A, PDCD10, TJP1, OCLN, HES1, MAPK1, VEGFA, EGFL7, NF1, and ENG, which are thought to be critical regulators of CCM pathology.^165^ They also preliminarily examined the potential roles of snoRNAs in three CCM specimens, identifying 271 differentially expressed snoRNAs.^166^ Following in situ hybridization validation, SNORD115‐32 and SNORD114‐22 were significantly decreased.

Exosomes are nanoscale vesicles secreted by cells containing diverse components, including DNA, ncRNA, proteins, metabolites, and lipids, and can stably exist in various body fluids. Li et al.^167^ collected blood samples from 14 patients with AVM and 14 healthy volunteers, identifying 117 dysregulated exosomal lncRNAs and 1159 dysregulated exosomal mRNAs. They further conducted an exosomal lncRNA–miRNA–mRNA‐related ceRNA regulatory network, identifying three significant modules involving 31 dysregulated exosomal lncRNAs, 114 dysregulated exosomal mRNAs, and KEGG pathways associated with Rap1, Ras, MAPK, and platelet activation. Another study, utilizing miRNA microarray analysis, found that miR‐3131, secreted by KRAS‐mutant ECs, can induce EndMT, with an increasing trend also observed in the peripheral blood of some patients with AVM.^105^ Given that EndMT is associated with the bleeding phenotype, this study suggests that exosomes may help predict the bleeding risk of CVMs in a noninvasive manner.

Renedo et al.^168^ analyzed blood samples from patients with AVM in the UK Biobank. They found that APOE ε4 status may identify patients with AVM at particularly high risk of intracerebral hemorrhage. Previous studies on the transcriptome, gut microbiota, and plasma proteome of CCM lesions have suggested that disease severity is linked to the brain–gut axis and proinflammatory responses. Srinath et al.^169^ employed liquid chromatography–mass spectrometry to evaluate the plasma metabolomics of patients with CCM and those with symptomatic bleeding. They found that plasma metabolites, including cholic acid and hypoxanthine, could distinguish patients with CCM, while arachidonic and linoleic acids differentiated those with symptomatic hemorrhage. These molecules are known to contribute to maintaining blood–brain barrier integrity, downregulating apoptosis, and alleviating inflammatory damage following hypoxia.^170^ These studies suggest a potential role for metabolites or lipids as biomarkers in CVMs, warranting further investigation.

### 4.3 Endovascular biopsy

An innovative approach to molecular profiling of CVMs without the need for open surgery was introduced by Winkler et al.^171^ through the use of endoluminal biopsy for the molecular profiling of human brain AVMs. They performed endoluminal biopsy and computational fluid dynamics (CFD) modeling in adults with unruptured AVMs during cerebral angiography. The endoluminal biopsies were successfully conducted in four patients without complications, revealing 106 differentially expressed genes that enriched pathogenic cascades, including RAS–MAPK signaling. The findings strongly correlated with genome‐wide expression data from surgically excised tissues. CFD analysis correlated wall shear stress with inflammatory pathway upregulation, and comparisons of pre‐ and postembolization samples confirmed flow‐mediated gene expression changes. This study illustrated that endoluminal biopsy enables molecular profiling of AVMs in living patients, identifying potentially targetable RAS–MAPK signaling abnormalities and facilitating precision medicine approaches.

These advances in biopsy techniques, encompassing cfDNA, ncRNAs, exosomes, and metabolites, highlight the potential of these technologies to revolutionize the diagnosis, monitoring, and treatment of CVMs. By providing noninvasive or minimally invasive means to profile genetic and molecular changes, these approaches facilitate early detection, dynamic monitoring, and the identification of targeted therapies. Although liquid biopsy offers a noninvasive method for identifying genetic mutations, it has inherent limitations, including the risk of misdiagnosis due to biomolecules from non‐CVM sources, leading to potential false positives. Similarly, endovascular biopsy poses procedural risks such as vascular injury and inflammation, which can complicate treatment outcomes.

## 5 TREATMENT OF CVMs

The paradigm shift in our understanding of CVMs, transitioning from purely structural anomalies to genetically and molecularly driven pathologies, has opened new avenues for therapeutic intervention. This evolving perspective has prompted the exploration of targeted therapies and immunomodulatory strategies to address the underlying causes of CVMs.

### 5.1 Targeted therapies

Targeted therapies have emerged as a promising treatment approach, given the identification of specific genetic mutations that drive CVMs. These therapies aim to inhibit molecular pathways activated by these mutations, thereby preventing disease progression and reducing the risk of complications. The drugs used to treat CVMs may target different aspects of CVM progression and rupture to provide therapeutic benefits. Current studies on AVMs are mainly based on in vitro proof‐of‐concept studies, with drug targets primarily focused on the KRAS/BRAF–MAPK, VEGF, and mural cell pathways.

Although KRAS mutations exist in many sporadic AVMs, the lack of direct KRAS inhibitors has led to the use of small‐molecule MEK inhibitors such as U0126 and SL327. These inhibitors, originally used to treat cancer, have shown efficacy in alleviating the phenotype in vitro and KRAS^G12V^ zebrafish models.^63^, ^97^ In another in vitro study, researchers used human umbilical vein ECs engineered to overexpress the MAP2K1^K57N^ mutation rather than the KRAS mutation to study pathway effects. They found that trametinib reduced ERK pathway activation and reversed vascular network formation, revealing its potential therapeutic effects in AVMs.^172^ Furthermore, Park et al.^98^ constructed KRAS^G12V/bEC^ mice and verified that trametinib effectively inhibited AVM growth and improved behavioral defects in this model.

In a BRAF^fl/V600E^ animal model, Tu et al.^100^ initiated dabrafenib treatment on the first‐day postinduction and continued the treatment for 6 weeks. They found that targeted inhibition of BRAF^V600E^ prevented AVM formation but did not rescue existing AVMs. Given that AVM lesions often develop fully before clinical treatment, dabrafenib may not be clinically effective. A prospective phase II open‐label pilot clinical trial is underway in Canada (NCT06098872), involving participants preparing for AVM surgical resection without a control group. Enrolled patients will receive trametinib orally once daily for 60 days before surgery, with the primary outcome being AVM imaging volume after the last dose.

Another crucial drug target for AVMs is the VEGF signaling pathway. Elevated levels of VEGF have been validated in various studies from plasma samples to specimens, particularly in high‐flow AVMs.^173^ Using an ALK1^fl/fl^ mouse AVM model, Cheng et al.^174^ found that elevated VEGF levels increased bleeding and mortality in mice. They further observed that the administration of bevacizumab, a humanized monoclonal anti‐VEGF antibody, reversed abnormal vascular density and dysplasia in the mouse mode.^175^ Seebauer et al.^173^ treated three adult patients with sporadic extracranial AVMs off‐label with bevacizumab, resulting in improved lesion deformity, although some side effects were noted.

Abnormalities in mural cells of AVMs are primarily related to hemodynamics, specifically manifested by decreased pericyte number and density. Pericyte coverage is reduced in ruptured AVMs, and a decreased pericyte number correlates with the severity of microbleeds and blood flow in the lesions.^104^ In the ALK1^fl/fl^ AVM mouse model, blood vessels exhibited increased diameter and loss of surrounding pericytes.^176^ Zhu et al.^177^ found that thalidomide, an immunomodulatory drug, reduced hemorrhage in this model.

For CCM, drug targets primarily include MEKK3–KLF2/4, RhoA/ROCK, PI3K/AKT/mTOR, and superoxide dismutase mimetics (Table 4). Defective ECs lacking any of the three CCM proteins exhibit overactivation of MEKK3–KLF2/4, which plays a crucial role in disease pathology. Ponatinib inhibits MEKK3 activity and normalizes downstream KLF expression in animal models, thereby inhibiting the formation of new CCM lesions and reducing the growth of established lesions.^178^ TSP1 and TGFβ/BMP, significant downstream targets of KLF2/4, are involved in CCM pathogenesis. Direct intervention with these targets can reverse the CCM phenotype both in vitro and in vivo.^117^, ^121^, ^179^

**TABLE 4:** Compounds and drugs tested for the treatment of CCM.

Study type and drug	Targeted molecular pathway	Effective in model	Source, year
In vitro and in vivo			
Ponatinib	MEKK3–KLF2/4	siCCM1 HUVECs	Choi et al.^178^
		CCM1iECKO; CCM2iECKO mice	
TSP1	TSP1	CCM1ECKO mice and derived BMEC	Lopez‐Ramirez et al.^121^
3TSR	TSP1	CCM1ECKO mice and derived BMEC	
DMH1	TGFb/BMP	CCM2iECKO mice	Maddaluno et al.^117^
LY364947	TGFb/BMP	CCM2iECKO mice	
SB431542	TGFb/BMP	CCM2iECKO mice	
Sulfide/sulindac sulfone	TGFb/BMP	CCM3iECKO mice	Bravi et al.^179^
Simvastatin	RhoA	siCCM2 HMVECs	Whitehead et al.^180^
		CCM2^+/tr^ mice	
		CCM1^+/−^ Msh2^−/−^; CCM2^+/−^ Msh2^−/−^ mice	Shenkar et al.^185^
		CCM3^+/−^ Trp53^−/−^; CCM3^+/−^ Msh2^−/−^ mice	Shenkar et al.^186^
Atorvastatin	RhoA	CCM3^+/−^ Trp53^−/−^; CCM3^+/−^ Msh2^−/−^ mice	
Fasudil	ROCK	CCM3^+/−^ Trp53^−/−^; CCM3^+/−^ Msh2^−/−^ mice	
		CCM2^+/−^ mice	Stockton et al.^187^
		CCM1^+/−^ Msh2^−/−^ mice	Shenkar et al.^185^
C3 transferase	ROCK	siCCM2 HMVECs	Whitehead et al.^180^
Y‐27632	ROCK	siCCM2 HMVECs	
		shCCM1/2/3 ECs	Borikova et al.^183^
BA‐1049	ROCK	CCM3^+/−^ Trp53^−/−^; CCM1^+/−^ Msh2^−/−^ mice	McKerracher et al.^184^
Rapamycin	mTORC1	siCCM1 HBMECs	Marchi et al.^188^
Torin1	mTORC1	siCCM1 HBMECs	
Rapamycin	mTORC1	CCM1iBECKO mice	Ren et al.^83^
Sorafenib	ERK	siCCM1 HUVECs	Wüstehube J, et al.^189^
NVP	EphB4	siCCM3 HUVECs	You et al.^190^
SU5416	VEGFR2	CCM3iECKO mice	He et al.^191^
		CCM1iECKO mice	DiStefano et al.^122^
Rebastinib	VEGFR2	CCM3iBECKO mice	Zhou et al.^120^
Propranolol	β‐Adrenoceptor receptor	CCM3iECKO mice	Oldenburg et al.^196^
Drug‐screening platforms			
REC‐994 (tempol)	SOD mimetic	CCM2iECKO mice	Gibson et al.^192^
Vitamin D3	Potential effect on inflammation	CCM2iECKO mice	
Ridaforolimus	mTOR	shCCM2 HUVECs; CCM1/2/3 mutant worm; CCM1 or CCM2 mutant Zebrafish	Otten et al.^193^
ENMD‐2076	FLT3 angiogenesis inhibitor	shCCM2 HUVECs; CCM1/2/3 mutant worm; CCM1 or CCM2 mutant Zebrafish	
DL‐erythro‐dihydrosphingosine	PKC; phospholipase A2 inhibitor; phospholipase D inhibitor	shCCM2 HUVECs; CCM1/2/3 mutant worm; CCM1 or CCM2 mutant Zebrafish	
DL‐homatropine hydrobromide	Muscarinic acetylcholine receptor agonist	shCCM2 HUVECs; CCM1/2/3 mutant worm; CCM1 or CCM2 mutant Zebrafish	
13‐cis‐retinoic acid	Anti‐inflammatory and antitumorigenic	shCCM2 HUVECs; CCM1/2/3 mutant worm; CCM1 or CCM2 mutant Zebrafish	
Clinical studies			
Propranolol	β‐Adrenoceptor receptor	Symptomatic CCM patients	Reinhard et al.^194^
		Symptomatic CCM patients	Zabramski et al.^195^
Atorvastatin	RhoA	Symptomatic CCM patients	Polster et al.^198^
Simvastatin	RhoA	CCM1 patients	Mabray et al.^197^

Abbreviations: EC, endothelial cells; HUVEC, human umbilic vein endothelial cells; HBMEC, human brain micro vascular cell.

The Rho/ROCK signaling pathway is abnormally activated when CCM proteins are inactivated, intersecting with the MEKK3–KLF2/4 signaling pathway, but appears to be downstream of MEKK3.^116^ Studies on mouse and human CCM lesions have shown that RhoA and its downstream effector ROCK1/2 are pathologically activated, leading to phosphorylation of myosin light chain (MLC) and MLC phosphatase and inhibition of MLC phosphatase catalytic activity.^180^, ^181^, ^182^ Initially, researchers observed that Rho/ROCK inhibitors Y‐27632 and C3 transferase reduced JNK activation, blocked the stress fiber response, and rescued endothelial barrier function in vitro.^180^, ^183^ Mice treated with BA‐1049, a novel ROCK 2 selective inhibitor, exhibited a significant dose‐dependent reduction in lesion volume and bleeding around the lesions.^184^ In a CCM2 mice model pretreated with simvastatin, an HMG‐CoA reductase inhibitor, the permeability response to VEGF was significantly reduced.^180^ Shenkar et al.^185^, ^186^, ^187^ further demonstrated that fasudil, high‐dose atorvastatin, and simvastatin significantly reduced chronic bleeding in CCM lesions, with fasudil and high‐dose atorvastatin being more effective than simvastatin in improving survival and slowing lesion development in vivo. PI3KCA overactivation is commonly observed in CCM lesions, leading to abnormal cell proliferation and symptomatic bleeding. Marchi et al.^188^ utilized rapamycin and other mTORC1 inhibitors, such as torin1, and found that they could slow the progression of CCM lesions in vivo and in vitro.^83^ Several studies have focused on the biological process of CCM‐related angiogenesis, proposing promising therapeutic targets, including ERK, EphB4, and VEGFR2 inhibitors.^120^, ^122^, ^189^, ^190^, ^191^

For noninvasive CCM treatments, some researchers have innovatively used data‐driven, unbiased, small‐molecule drug screening and ML platforms.^192^, ^193^ Gibson et al.^192^ conducted automated immunofluorescence and ML‐based preliminary screening of the structural phenotype of CCM2‐deficient human ECs and further screening based on changes in endothelial function and animal model outcomes. They ultimately screened 2100 known drugs and bioactive compounds, identifying vitamin D3 and tempol as candidate drugs.^192^ Otten et al.^193^ applied 5268 substances to CCM‐mutant worms, zebrafish, mice, and human ECs. Their findings were integrated with a whole transcriptome profile of zebrafish CCM2 mutants using a systems biology‐based target prediction tool to reveal disease‐related signaling pathways and potential targets for small‐molecule‐based therapeutics.

In clinical studies, research on effective drugs for CCM began with a series of case reports on propranolol.^194^, ^195^ Based on this, preclinical studies conducted by Oldenburg et al.^196^ suggested that propranolol may help reduce and stabilize vascular lesions, positioning it as a potential medication for CCM. Mabray et al.^197^ conducted a small prospective randomized controlled trial pilot study (NCT01764451) to evaluate the safety of simvastatin in patients with CCM and the feasibility of using imaging measures for efficacy assessment. However, the study was unable to draw reliable conclusions regarding its efficacy. A phase I/IIa placebo‐controlled, double‐blind, single‐center clinical trial is currently underway (NCT0176445) to investigate the effectiveness of atorvastatin for treating symptomatic bleeding in CCM.^198^ The treatment group will receive 80 mg of atorvastatin orally daily as the starting dose, while the control group will receive a placebo. CCMs showing symptomatic bleeding within the past year will be evaluated by MRI quantitative magnetic susceptibility mapping to determine whether atorvastatin produces a significant difference in lesional iron deposition compared with the placebo. However, the results of this study have not yet been published.

### 5.2 Immunotherapy

Identifying immune components in CVM pathogenesis has spurred interest in immunotherapy as a potential treatment modality. Studies have shown that immune cells, particularly macrophages, are crucial in the development and progression of CVMs.^101^, ^199^ Furthermore, immune cell infiltration correlates with lesion progression, suggesting that targeting these immune cells or modulating their activity may provide a novel approach to disease management.

Chen et al.^200^ found a significant increase in neutrophils and macrophages in AVM tissues, while T lymphocytes and B lymphocytes were infrequently observed. The study identified myeloperoxidase (MPO) levels as a potential indicator to evaluate neutrophil count; a higher neutrophil count in AVMs corresponded to elevated MPO and matrix metalloproteinase‐9 (MMP‐9) levels. Weinsheimer et al.^201^ characterized the blood transcriptional profiles of patients with AVM, and found that 29 genes were differentially expressed between patients with unruptured AVM and controls, with 13 potentially predictive of AVM. Another study found that IL‐6 levels in the blood and activated MMP‐9 in the tissues of patients with ruptured AVM were significantly higher than those in the nonruptured and control groups, suggesting a potential role of inflammation in AVM pathogenesis.^202^ Noshiro et al.^203^ examined 18 sporadic AVM specimens and found that the mRNA expression of IL‐6 was also significantly higher than that in control tissues. Notably, macrophages and other inflammatory cells are frequently observed, even in AVMs without a history of rupture, indicating their intrinsic role in AVM pathogenesis rather than merely a response to hemorrhage.^204^, ^205^ Winkler et al.^171^ classified cerebrovascular‐related immune cells at the single‐cell level, identifying 17 cell subsets, including nine myeloid cell subsets, dendritic cells, glial cells, three perivascular macrophage subsets (pvMφ), and three monocyte (Mo) subsets. Myeloid immune cells constitute a significant proportion of AVMs, indicating immune activation during the disease process.^171^ In contrast, a single‐cell resolution molecular atlas of human cerebral vasculature recently published in *Nature* showed that the proportion of myeloid cells in AVMs was lower than that in control tissues.^101^ These findings indicate that the components of the immune microenvironment in AVMs warrant further investigation at the single‐cell level. Given that both ruptured and unruptured AVMs are associated with inflammatory processes, researchers have rationally targeted this process by using anti‐inflammatory drugs in preclinical models. Treatment with the MMP‐9 inhibitors minocycline and pyrrolidine dithiocarbamate reduced brain MMP‐9 activity and attenuated VEGF‐induced stroke in a mouse model.^206^ Similarly, doxycycline lowered MMP‐9 levels in the same mouse model.^207^ The effects of doxycycline were also explored in patients with AVMs before surgical resection. However, due to the short duration of medication and the small patient sample, only a trend toward reduced MMP‐9 levels was observed, which did not reach statistical significance.^208^ Antiangiogenic drugs, such as bevacizumab, have been shown to reduce vascular density and dysplasia in a mouse model of brain AVM.^175^ Furthermore, thalidomide reduced the burden of CD68+ cells and the expression of inflammatory cytokines in AVM lesions, although potential side effects cannot be ignored.^177^

Regarding the immune microenvironment of CCM, the Kahn laboratory at the University of Pennsylvania reported a novel and intriguing role of the gut–brain axis in CCM pathogenesis. In a CCM mouse model, lipopolysaccharides activated the TLR4 receptor on ECs and its downstream MEK5–ERK5–KLF2/4 pathway, promoting CCM development while knocking out the TLR4 receptor significantly reduced CCM lesion formation.^126^ Given the critical role of TLR4 in CCM pathophysiology, TLR4 antagonists (LPS‐RS) may serve as potential therapeutic agents for CCM. In a subsequent mouse model, the team increased CCM formation after chemically disrupting the intestinal barrier with sodium dextran sulfate, suggesting that the intestinal barrier is the primary determinant of CCM progression rather than the microbiome.^127^ They further revealed the combined effects of dexamethasone on both brain ECs and intestinal epithelial cells, suggesting that dexamethasone could effectively inhibit CCM formation in mice. Previous studies have also reported that TLR4 and Fc receptors (FcR) can synergistically activate common downstream molecules, producing cumulative effects.^209^ TLR4 and FcR expression in CCM ECs is significantly higher than that in normal cerebral vascular ECs (Cao et al., unpublished data).

In CCM, mutant ECs can reduce intercellular junction protein expression and destroy the CCM blood–brain barrier. Mutant ECs can also recruit inflammatory and immune cells by overexpressing immune adhesion chemokines, promoting the CCM immune microenvironment. Mutated ECs lining blood vessels are in close proximity to immune cells in circulation, leading to an accumulation of myeloid cells, lymphocytes, and other immune cells around CCM lesions.^123^, ^210^, ^211^, ^212^, ^213^ Shi et al.^214^ suggested that plasma cells, differentiated from activated mature B cells, exist in CCMs, with IgG and complement membrane attack complexes co‐localizing in CCM lesions. The IgG in CCM lesions amplifies in an oligoclonal pattern, suggesting that this amplification was antigen‐specific.^215^ Furthermore, Shi et al.^214^ sequenced B cells obtained via laser microdissection of tissue specimens, revealing the clonal expansion of in situ B cells, which suggests specific antigen stimulation occurs in CCM. Zhang et al.^216^ analyzed laser‐captured microdissected plasma cells. They found that antibodies produced by plasma cells in CCM lesions typically target cytoplasmic and cytoskeletal autoantigens, including NMMHC IIA, vimentin, and tubulin.^216^ Given that plasma cell‐mediated immunity may play a role in CCM lesions, studies have examined the effects of B cell knockout on imaging findings on CCM lesions. Research has shown that nonheme iron deposition and ROCK activity are reduced in the lesions of B cell‐depleted mice, suggesting the potential application of B cell inhibitors in CCM treatment.^217^ Mechanisms such as neutrophil extracellular trapping and macrophage inflammatory infiltration have also been validated in clinical specimens, opening new avenues for research in this field.^148^, ^218^, ^219^, ^220^, ^221^

## 6 FUTURE DIRECTIONS

**FIGURE 3:**
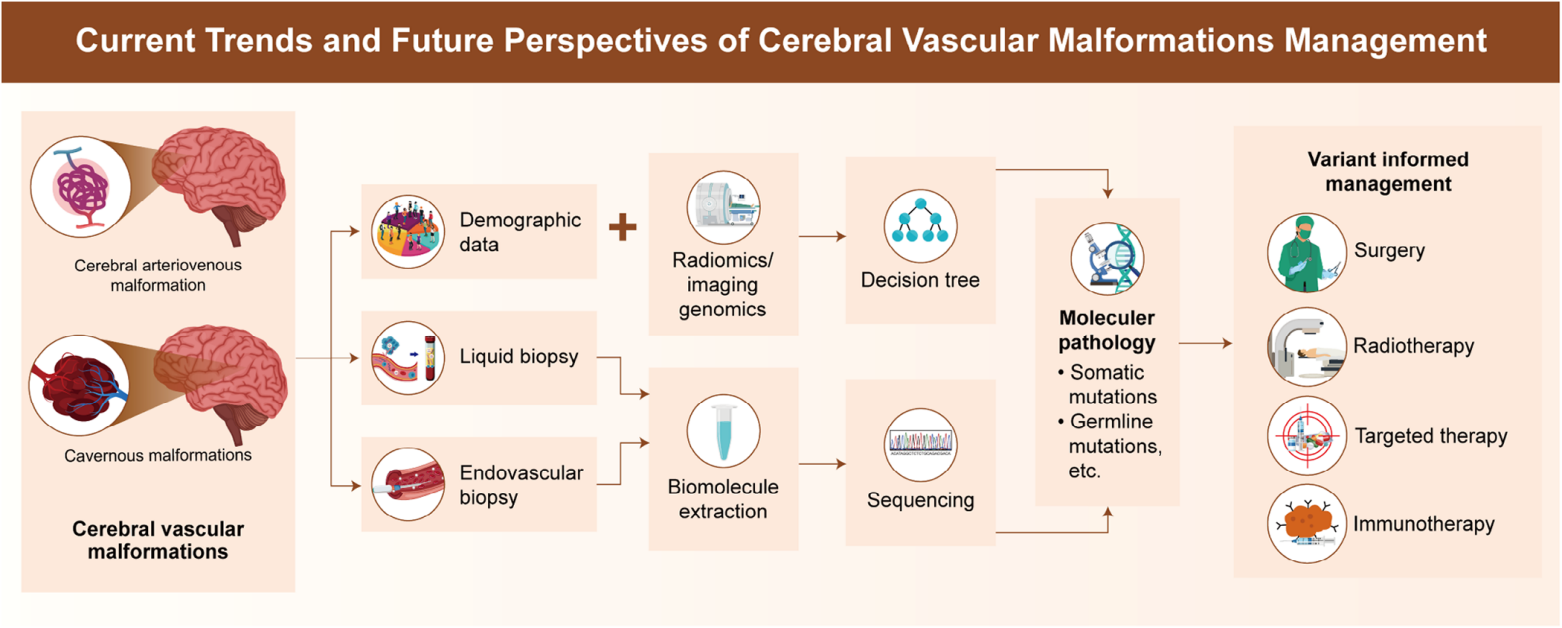
CVMs management based on imaging genomics and liquid biopsy. In the future, somatic or de novo germline mutation information could be used to direct neurosurgical and medical management of CVMs. Somatic or germline variants can be acquired from imaging genomics or liquid biopsies preoperatively. These variants could then be correlated with clinical demographics and medical history to expand research efforts and identify targeted therapies or immunotherapy that could be used as monotherapy or adjuncts to surgery. This information could also be used for surgical planning if necessary.

The evolving landscape of CVM research and treatment has deepened our understanding of its pathogenesis. Integrating genetic, molecular, and immune insights into clinical practice can revolutionize the management of CVMs, leading to a more personalized and precise approach to patient care (Figure 3).

### 6.1 Future in imaging genomics

Imaging genomics is still in its early stages, with most studies focusing on identifying novel imaging markers. The future lies in developing big‐data analytics and AI‐driven methods. A key objective is establishing a link between genetic mutations and imaging features to better predict patient prognosis. Future research should also prioritize developing standardized protocols for imaging genomics to ensure consistency and reproducibility across research and clinical settings. Additionally, integrating AI and ML algorithms will enhance the diagnostic accuracy and predictive capabilities of imaging genomics, facilitating early detection and personalized treatment strategies.

### 6.2 Role of liquid or endovascular biopsy

The future of CVM treatment lies in precision medicine, which tailors therapeutic interventions to a patient's genetic and molecular profile. Advances in liquid biopsy will play a crucial role in realizing this goal. By identifying specific genetic mutations and molecular pathways involved in individual patient, clinicians can select targeted therapies that are effective, minimizing unnecessary treatments and optimizing outcomes.

In 2023, Fan et al.^222^ conducted a nontargeted metabolite detection analysis of patient plasma, revealing significant changes in the metabolic profiles of patients with extracranial AVMs. This highlights metabolomics as a valuable diagnostic tool for CVMs. Similarly, lipidomics offers a new dimension to liquid biopsy approaches, providing insights into lipid alterations underlying CVMs. Future studies should explore integrating metabolomics, lipidomics, and other liquid biopsy approaches, such as circulating ncRNA and exosome analysis, to develop comprehensive biomarker panels for the early diagnosis and monitoring of CVMs.

### 6.3 Advancing targeted therapies

Recent advancements in targeted therapies have shown promise in treating CVMs. In patients with tumors, research on upstream inhibitors such as direct KRAS^G12C^ or KRAS^G12D^ inhibitors is emerging. Fraissenon et al.^223^ demonstrated the effectiveness of sotorasib, a specific KRAS^G12C^ inhibitor, in reducing vascular malformation volume and improving survival in two mouse models with mosaic KRAS^G12C^. Additionally, two adult patients with KRAS^G12C^‐associated AVMs showed rapid symptom and lesion size reduction after sotorasib treatment. However, similar drugs targeting KRAS^G12D^ have yet to be developed and validated for brain CVMs. Future research should focus on developing and testing these inhibitors in clinical trials to evaluate their efficacy and safety.

### 6.4 Exploring the immune microenvironment

Understanding the role of the immune microenvironment in CVM pathogenesis has spurred interest in immunotherapy as a potential treatment modality. Tertiary lymphoid structures (TLSs), known for their role in rapid and efficient T cell‐dependent B cell activation, have gained attention and have been reported to be ectopic around chronic inflammatory sites in diseases, such as those involving tumors, infections, autoimmune diseases, and organ transplantation.^224^, ^225^ TLSs promote B cell maturation, antibody subtype conversion, and somatic hypermutation. After clonal type screening and affinity maturation, B cell clones differentiate into plasma cells to produce specific IgGs targeting specific antigens.^226^, ^227^ However, this phenomenon has not been reported in CVMs.

Future research should investigate the presence and potential role of TLSs in CVMs and investigate targeting these structures for therapeutic purposes.

### 6.5 Addressing challenges and improving outcomes

Despite significant progress in understanding and treating CVMs, several challenges remain unresolved. The genetic and clinical heterogeneity of CVMs complicates the development of universally effective therapies. Additionally, the long‐term safety and efficacy of novel treatments must be rigorously evaluated, particularly those targeting genetic and immune pathways. Ensuring new treatments are safe and effective across diverse patient populations is crucial for their successful implementation in clinical practice.

Collaboration among researchers, clinicians, and pharmaceutical companies is essential for translating these advancements into clinical practice. Large‐scale multicenter clinical trials are necessary to validate the efficacy and safety of emerging therapies. Continued investment in innovative diagnostic and therapeutic approaches is critical to improving outcomes for patients with CVMs.

## 7 CONCLUSION

CVM research is undergoing a transformative shift driven by genetic and molecular research advances. The future is promising, with significant advancements in imaging genomics, liquid biopsy, targeted therapies, and immunotherapy. By integrating these cutting‐edge technologies and approaches, the field is moving toward a more personalized and precise approach to patient care. Continued research and collaboration are essential to overcome these challenges and realize the full potential of these advancements, ultimately improving patient outcomes.

## AUTHOR CONTRIBUTIONS

Qiheng He and Yong Cao were involved in study conception and design. Ran Huo, Yingfan Sun, Zhiyao Zheng, Hongyuan Xu, Shaozhi Zhao, Yang Ni, Qifeng Yu, Yuming Jiao, and Wenqian Zhang collected the literature. Qiheng He summarized the literature. Qiheng He, Yingfan Sun, and Zhiyao Zheng wrote the original manuscript. Jizong Zhao and Yong Cao supervised the study. All authors reviewed and approved the manuscript for publication.

## CONFLICT OF INTEREST STATEMENT

The authors declare no conflict of interest.

## ETHICS STATEMENT

Not required.

## Supporting information



Supporting Information

## Data Availability

Not required.
