# Supplementary material for: Cerebral vascular malformations: pathogenesis and therapy
Source: MedComm (2020). 2024 Dec 8;5(12):e70027. doi: 10.1002/mco2.70027 (PMC11625509; doi:10.1002/mco2.70027)
Supplement: Supplementary file 1 — Supporting Information [file MCO2-5-e70027-s001.docx]

***Supplementary Materials***

**Cerebral Vascular Malformations: Pathogenesis and Therapy**

Running title: AVM and CCM: Pathogenesis and Therapy

Qiheng He^1, 2^, Ran Huo^1, 2^, Yingfan Sun^1, 2^, Zhiyao Zheng^1, 4, 5^, Hongyuan Xu^1, 2^, Shaozhi Zhao^1, 2^, Yang Ni^1^, Qifeng Yu^1, 2^, Yuming Jiao^1, 2^, Wenqian Zhang^1, 2^, Jizong Zhao^1, 2*^, Yong Cao^1, 2, 3*^

Affiliations:

^1^ Department of Neurosurgery, Beijing Tiantan Hospital, Capital Medical University, Beijing 100070, China

^2^ Center for Basic and Translational Medicine, China National Clinical Research Center for Neurological Diseases, Beijing 100070, China

^3^ Academic Committee, Beijing Institute of Brain Disorders, Beijing 100069, China

^4^ Research Unit of Accurate Diagnosis, Treatment, and Translational Medicine of Brain Tumors Chinese Academy of Medical Sciences and Peking Union Medical College Beijing China

^5^ Department of Neurosurgery Peking Union Medical College Hospital, Chinese Academy of Medical Sciences and Peking Union Medical College Beijing China

***Corresponding author:**

Yong Cao, caoyong@bjtth.org

Jizong Zhao, zhaojizong@bjtth.org

**Supplementary Table**

| **Table S1.** Clinical studies on liquid biopsy and endovascular biopsy. | | | |
| --- | --- | --- | --- |
| Study, year | Type | Biomolecule | Conclusion |
| AVM |  |  |  |
| Serio et al, 2022 | cfDNA | KRAS: 8 in 15 patients FGFR2: 1 in 15 patients KDR: 1 in 15 patients GLMN: 1 in 15 patients MAP2K1: 2 in 15 patients FGFR3: 1 in 15 patients | The study demonstrates power of cfDNA-NGS liquid biopsy in vascular malformations clinical classification, diagnosis and treatment. |
| Zenner et al, 2021 | cfDNA | MAP2K1: 7 in 8 patients BRAF: 1 in 8 patients | Variants were detected in plasma and cyst fluid from VM patients,providing opportunities to initiate targeted pharmacotherapies without prior surgery . |
| Li et al, 2018 | lncRNA | ENST00000423394, ENST00000444114, TCONS_00013855, ENST00000452148 | The study discovered that development of AVM may correspond with downregulation of NADPH reductase, lipoprotein lipase and Optic atrophy related proteins. |
| Huang et al, 2017 | miRNA | miR-137 and miR-195* | This study discovered that miR-137 and miR-195* act as vasculogenic suppressors in AVMs by altering phenotypic properties of AVMSMCs, and that the absence of miR-137 and miR-195* expression leads to abnormal vasculogenesis. |
| Chen et al, 2018 | miRNA | miR-7-5p, miR-199a-5p, and miR-200b-3p | This is the first study to present the global miRNA  expression profiling in peripheral blood of BAVMs, providing an important foundation for future studies on the regulation of miRNAs involved in BAVMs. |
| Lee et al, 2024 | miRNA | miR-135b-5p | The study discovered that miR-135b-5p could affect the pathophysiological process of AVM and might play a vital role as a potential biomarker of AVMs for application related to diagnosis and treatment. |
| Li et al, 2020 | lncRNA, mRNA | MIR4435-1HG,  LINC00657,  LOC101927854,  SEPT5-GP1BB | This study identified dysregulated exosomal lncRNAs and mRNAs in AVMs, demonstrating the involvement of dysregulated lncRNA and mRNA patterns in AVMs and constructed an exosomal competitive endogenous RNA regulatory network. |
| He et al, 2023 | miRNA | miR-3131 | Exosomal miR-3131 promotes EndMT in KRAS-mutant bAVMs, and miR-3131 could be a potential biomarker and therapeutic target in KRAS G12D mutant bAVMs. |
| Winkler et al, 2022 | RNA | 106 differentially expressed genes | Endoluminal biopsy allows molecular profiling of bAVMs in living patients, and its integration with CFD allows determination of flow-mediated transcriptomic alterations. Endoluminal biopsy could help facilitate trials of precision medicine approaches to bAVMs in humans. |
| CCM |  |  |  |
| Subhash et al, 2019 | lncRNA | LBX2-AS1, SMIM25/LINC01272 | The study demonstrated that lncRNAs are prevalent in CCMs disease and are likely to play critical roles in regulating important signaling pathways involved in the disease progression,providing useful insights into the biology and, ultimately, contribute in preventing this debilitating disease. |
| Kar et al, 2017 | miRNA | let-7b-5p, miR-361-5p, miR-370-3p, miR-181a-2-3p and miR-95-3p | This study provided the first evidence that there are candidate  miRNAs crucial for CCM pathology, in which let-7b 5p, miR-361-5p, and miR-370-3p may be of biological and  functional relevance for patients suffering from CCM within  the brainstem. |
| Kar et al, 2018 | snoRNA | SNORD115-32,  SNORD114-22,  SNORD113-3 | Three snoRNAs were found to be significantly downregulated in CCM patients compared to healthy people. |
| Srinath et al, 2023 | miRNA | miR-486-5p, miR-25-3p, miR-16-5p, miR-183-5p, miR-501-3p,miR-182-5p, miR-20a-5p and miR-92a-3p | Plasma metabolites reflect CCMs and their hemorrhagic activity, and a model of their multiomic integration could be applicable to other pathologies. |
